# Modeling individual differences in vocabulary development: A large‐scale study on Japanese heritage speakers

**DOI:** 10.1111/cdev.14168

**Published:** 2024-09-29

**Authors:** Maki Kubota, Jason Rothman

**Affiliations:** ^1^ University of Bergen Bergen Norway; ^2^ Lancaster University Lancaster UK; ^3^ UiT The Arctic University of Norway Tromso Norway; ^4^ University of Nebrija Madrid Spain

## Abstract

This study examines when the vocabulary knowledge of Japanese heritage speakers (HSs; *N* = 427, *M*_age_ = 9.96, female = 213) begins to diverge from monolingual counterparts (*N* = 136, *M*_age_ = 6.69, female = 65) and what factors explain individual differences in HS development. Vocabulary of HSs began to diverge from 5.61 years and this difference lasted until they were young adults. We also administered a fit‐for‐purpose questionnaire in 2021–2023 and identified six experiential latent factors: Holiday, School, Community, Proficiency, Literacy, and Home. Structural modeling indicates that Holiday predicted vocabulary scores, while Holiday and Literacy predicted Proficiency. Our findings highlight the importance of immersion experiences and literacy engagement for heritage language development.

**Abbreviations**
CFA: confirmatory factor analysis
EFA: exploratory factor analysis
GAMM: generalized additive mixed modeling
HL: heritage language
HLB: heritage language bilingualism
HS: heritage speaker
ML: majority language
PVT–R: Picture Vocabulary Task Revised
Q‐bex: quantifying bilingual experience
SEM: structural equation modeling
SES: socioeconomic status

Acquiring a language is, in principle, an automatic and easy process—everyone who undergoes normal development learns to speak at least one language. However, while it is perfectly natural to acquire more than one language, the processes involved are more complex for bilingualism. After all, acquiring two languages entails learning twice the number of words and structures, often on the basis of considerably less exposure to one or both of the languages. Nevertheless, research consistently shows that despite young (simultaneous) bilingual children often displaying slight delays in mastering grammar and having smaller vocabularies in at least one language compared to monolingual peers, there are no significant differences in the developmental trajectory of their two languages (Byers‐Heinlein & Lew‐Williams, 2013; Meisel, 2011; Serratrice, 2013; see, e.g., Armon‐Lotem et al., 2021; Thordardottir, 2011 for literature on simultaneous vs. sequential bilinguals' vocabulary development in the heritage language [HL]). This observation is not surprising, considering the fact that the fundamental processes of language acquisition and the underlying mechanisms are the same irrespective of how languages are to be naturalistically acquired.

Contrary to the prevailing findings on the language development of early bilingual children, a substantial body of work indicates a different pattern in their eventual mastery of the HL in young adulthood. Heritage speakers (HSs) are bilinguals who acquire a minority or HL at home (or are otherwise readily available to them in early childhood), distinct from the majority societal language in which they grow up (Rothman, 2009). Although exceptions are documented (see Kupisch & Rothman, 2018), studies examining HL competence and performance outcomes in adult HSs often demonstrate a level of proficiency and use that differs from that of monolingual comparisons at the aggregate level (see Montrul, 2016; Polinsky, 2018; Polinsky & Scontras, 2020). Moreover, studies also show that the range of HS individual differences is considerable, much wider than any range of differences attested in monolingual speakers (Paradis, 2023). The typical adult outcomes of HSs thus raise significant questions considering the fact that HSs, like monolinguals, are exposed naturalistically to the HL as a first language (L1) from birth (Rothman & Treffers‐Daller, 2014). Why is heritage language bilingualism (HLB) characterized by such variation—from monolingual peers and across individual HSs—in language knowledge and use? What factors predict the associated developmental outcomes?

This study sets out to examine these questions by investigating the vocabulary knowledge of Japanese HSs and their monolingual counterparts by employing a newly established questionnaire tailor‐made to precisely document language experiences of HSs (De Cat et al., 2023). The large sample size of this study also allows us to control for covariates between monolinguals and bilinguals (such as socioeconomic status [SES] and family structure) and use structural equation modeling (SEM) to model complex relationships involving multiple dependent and independent variables as well as unobserved latent constructs. Unraveling the contributing variables and their relative significance that shape the spectrum of language outcomes in the HL is particularly crucial, given that more than half the global population is bilingual (Shook & Marian, 2012), and with people emigrating at increasingly higher rates year on year, HLB is on a sharp incline globally.

Although this has been changing in recent years as the field begins to recognize that, whether explicitly referred to as such, most subjects in early child bilingualism work qualify as (child) HSs (Kupisch & Rothman, 2018), the majority of studies using the label “heritage speaker” has historically examined language competence in young adults (typically undergraduate university students). For instance Montrul and Foote (2014), a highly relevant study given the domain of this study, examined the lexical access of late L2 learners of Spanish and HSs of Spanish (*M*_age_ 21.2 and 22.4, respectively) by employing a lexical decision task and a translation task. The results showed no difference in overall accuracy of lexical access between the two groups, and the late L2 learners demonstrated faster reaction times than the HSs. Moreover, the expected age in which the stimulus words were acquired (i.e., early or late acquired) predicted lexical access accuracy and speed among both groups, suggesting that the age in which words are encountered and language experience determine lexical processing of the nondominant language (Spanish) for late L2 learners and HSs. Dubiel and Guilfoyle (2021), presenting a study with a more comparable age range to that of the present one, tested the lexical access of Polish monolingual and Polish–English HS children (ages 4.7–13.2) via a picture‐naming task and also found no differences in accuracy between the two groups. However, the reaction time was slower across all age spans, indicating slower language access for the HS children.

The outcomes of these studies consistently demonstrate differences between HSs and L1‐dominant participants (most typically functional monolinguals groups), confirming that HSs' language outcomes are often distinct. Yet, these data do not provide insights into how or why the differences arise. This is the case despite all current hypotheses proposing that such differences result from factors influencing HL development during the transition from middle childhood to early adulthood (following the onset of school age). Regardless of whether these factors are reducible to arrested development, attrition, input delimitation, sociodemographic variables, or some combination thereof, testing such claims remains challenging due to the existing focus on outcomes rather than the developmental process itself. Nonetheless, in recent years, there has been an upsurge of studies that focus on examining factors that modulate individual differences in the HL of children for grammar and vocabulary (Armon‐Lotem et al., 2021; Bayram et al., 2019; Chondrogianni & Schwartz, 2020; Daskalaki et al., 2019; Flores et al., 2017; Hao & Chondrogianni, 2021; Meir & Janssen, 2021; Mitrofanova et al., 2022; Torregrossa et al., 2023; van Osch et al., 2019). Among those that focused on vocabulary development in HS children, small‐ to midscale studies employing simple correlational or regression analyses find a relationship between lexical diversity and age of onset to the majority language (ML; Gharibi & Boers, 2019), expressive vocabulary and input and output quantity at home and the number of parents who speak the HL (Correia & Flores, 2017), receptive vocabulary and SES (Li et al., 2021; Montanari et al., 2020), expressive vocabulary and the amount (in hours) of exposure to HL (DeVellis & Thorpe, 2021), and visits to the homeland and expressive vocabulary (Chondrogianni & Daskalaki, 2023).

Particularly relevant work comes from a large‐scale study by Sun et al. (2020) with 457 child HSs (ages 5.25–6.58) who either spoke Mandarin, Malay, or Tamil as a HL in Singapore (i.e., English as the ML). They found that experience‐related factors such as HL input at home, number of books in the HL, the amount of HL media exposure, and the length of time that the children spent at kindergarten predicted children's receptive HL vocabulary, while internal factors such as language combination, SES, nonverbal intelligence, or working memory did not. Their subsequent study (Sun et al., 2022) with 201 HSs of Mandarin Chinese in Singapore (ages 4–5) also found that the number of books in the HL, the number of contexts in which only HL is spoken, the mother's HL proficiency, and age of onset to the HL predicted HL receptive vocabulary after controlling for SES, gender, nonverbal intelligence, and phonological working memory.

Although the findings of these large‐scale studies make significant contributions by having adequate statistical power to predict vocabulary development, they have mainly examined children from narrow age ranges (including ages 5–6.4, *N* = 240 for Armon‐Lotem et al., 2021), and thus it is still unclear when the divergence between monolinguals and HSs begins to emerge from childhood to adolescence, much less than its path over time. Moreover, as pointed out by the authors themselves, Sun et al. (2020) suggest that there needs to be better questionnaires that precisely capture the language experiences (especially language exposure) of HSs in various contexts.

This study further contributes to the field by targeting HSs from a single language, Japanese, in two linguistic environments—English and German dominant—from a considerably wider age range. Doing so not only allows us to pinpoint the age at which vocabulary knowledge starts to significantly differ, but also permits a comparison of HL developmental trajectories to that of their monolingual peers (while matching them on covariate factors) over the crucial time period between early childhood and young adulthood. We specifically examined HL development of HSs in German‐dominant and English‐dominant environments since our secondary objective was to examine cross‐linguistic effects of grammatical properties (e.g., overt case marking, shared by German but not English). Since the current investigation focuses on vocabulary development, we did not expect any cross‐linguistic effects from German and/or English to Japanese. Rather, as we explore in this article, we expected experiential factors to predict HL vocabulary development.

Moreover, we use a recently established questionnaire that is specifically tailored to uncover the language experiences and characteristics of HSs (quantifying bilingual experience [Q‐bex]; De Cat et al., 2023). We utilize exploratory factor analysis (EFA) and SEM to extract latent factors to predict vocabulary knowledge, rather than entering single questionnaire responses in the model (as done with most studies described earlier), which can be more prone to measurement errors and multicollinearity. Given this context, our confirmatory study asked the following research questions:

1. When does the vocabulary knowledge of HSs begin to diverge from their monolingual counterparts (all things being equal in terms of the age, gender, number of siblings, and main caretaker's education)?

According to previous literature (e.g., Montrul, 2010; Sun et al., 2020), we predict that receptive vocabulary knowledge will begin to diverge around 5–7 years old—the general age in which children enter the school system and their language dominance in exposure and use starts to shift toward the societal ML.

2 What are the underlying latent factors that can be extracted from the Q‐bex questionnaire?

We expect the Q‐bex questionnaire to extract factors related to HL use and exposure at home, school, society, as well as literacy skills and family structure (such as SES, number of siblings, and number of adults living in the home).

3 What bilingual constructs predict children's vocabulary knowledge in their HL?

In line with previous studies (e.g., Correia & Flores, 2017; DeVellis & Thorpe, 2021; Sun et al., 2022), we expect language exposure and use at home as well as literacy engagement to predict children's HL vocabulary.

## METHOD

### Participants

We initially collected data from 145 monolingual and 457 HS participants from 2021 to 2023. However, we excluded those (a) not meeting the age criteria (see below), (b) indicating language impairment or developmental disorders, (c) missing questionnaire or vocabulary score data, (d) not exposed to the Japanese before the age of 2 (for HSs only), (e) not having resided in the English or German ML context for at least two third of their life (for HSs only). According to these criteria, nine participants were removed from the monolingual sample and 30 participants from the HSs. Thus, the final participants in the study were 136 monolingual Japanese children (*M*_age_ = 6.69, range = 3.05–16.57, female = 65) and 427 Japanese HSs (*M*_age_ = 9.96, range = 4.02–18.18, female = 213; 313 from English‐dominant environment, 114 from German‐dominant environment). The monolingual children were all L1 speakers of Japanese living in Japan with Japanese parents. Their SES was measured via the main caretaker's final education from a scale of 1 to 5 (1 = none, 2 = primary school, 3 = secondary school or equivalent, 4 = postsecondary school training but not a university degree, 5 = university degree; *M* = 4.45, range = 3–5). The monolingual participants were recruited through a Japanese online recruitment platform (Lancers) and were compensated 1000 yen for their participation.

The HSs were all exposed to Japanese before the age of 2 (*M* = 0.93 months, SD = 3.85, range = 0–24). The majority of the HSs were second‐generation immigrants, and eight participants were third‐generation and two participants were fourth‐generation HSs. The HSs from an English‐dominant environment lived in the following countries: USA (*n* = 190), Australia (*n* = 45), Canada (*n* = 45), and United Kingdom (*n* = 33), and those from a German‐dominant environment came from Germany (*n* = 107) and Switzerland (*n* = 7). Their mean onset to the societal ML (English or German) was 11.75 months (SD = 18.53, range = 0–78 months). We only included children who attended schools in the ML of the society. The children's average length of exposure to the ML through schooling was 7.25 years (SD = 3.45, min = 0.32, max = 16.2). Their SES was also measured via the main caretaker's final education from a scale of 1 to 5 (*M* = 4.05, range = 1–5). The HS participants were recruited via personal networks, Japanese Saturday schools, Facebook groups, and Japanese communities abroad; they were compensated 10 euros or pounds or dollars for their participation.

### Materials

We administered a Picture Vocabulary Task Revised (PVT–R) in Japanese (Ueno et al., 2008) to all participants. In this task, the participants were presented with four pictures and were asked to choose the picture that best described the word provided to them in audio format. The PVT–R consisted of 90 trials with increasing difficulty, and it ended when the participant made three consecutive errors. The maximum trial number reached was used as a measure of their vocabulary score.

We collected detailed information of their language background experience using the Q‐bex questionnaire (De Cat et al., 2023). Q‐bex is a newly established, user‐friendly, online questionnaire that can be tailored in many ways to facilitate administration and ensure the desired level of detail is obtained. Q‐bex contains two modules which are obligatory (background information and risk factors) and another five modules (language exposure and use, language proficiency, richness of linguistic experience, attitudes and satisfaction with child's language, language mixing), which can be individually selected or excluded altogether to meet the individual needs of researchers or practitioners. Since administering the entire module takes more than an hour, we included specific submodules that we hypothesized to be the most important in the context of our study. These include: current estimates, cumulative estimates, age and place of first exposure, overheard speech at home, proficiency (no reference group), activities, caregiver's education, estimated diversity of speakers, preferred language, language mixing at home and outside between interlocutors.

### Procedure

**FIGURE 1:**
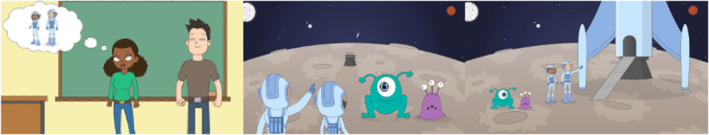
Example animations scenes from the experiment.

The data were collected remotely using the Gorilla experiment builder platform, thus participants took part in the study from their own homes. We ensured that they could only access the experiment from their laptops or computers and not their phones or iPads. Participants were first shown a general introduction video, instructing them to be in a quiet environment with no distractions. Parents were explicitly told not to provide answers to their children, and if the children were below 12 years old, they were asked to supervise them. After obtaining parental consent, the children watched a short animation cover story featuring two astronauts, Ken and Lisa, whose spaceship was stolen by aliens, see Figure 1. The children were tasked with helping Ken and Lisa to reclaim their spaceship by completing various missions involving different creatures. Participants completed a production task prior to the vocabulary task and a comprehension task after the vocabulary task. Once all tasks were completed, parents (with the child) were requested to fill out the language background questionnaire (Q‐bex) and a compensation form. The entire online experiment can be accessed through the Gorilla open materials page using the following link: https://app.gorilla.sc/openmaterials/686845.

## RESULTS

### When does the vocabulary knowledge of HSs begin to diverge from their monolingual counterparts (all things being equal in terms of family structure, SES, and gender)?

The descriptive analysis showed that the mean vocabulary scores for monolinguals was 46.36 (SD = 26.71, range = 0–90) and 45.28 (SD = 22.64, range = 0–90) for the HSs. In order to examine when the development of vocabulary knowledge begins to diverge between monolinguals and bilinguals and how long this difference lasts, we first used the MatchIt function in R (Ho et al., 2007) to create balanced comparison groups between monolinguals and HSs, matching them based on their observed characteristics or covariates, which included age, SES, gender, and the number of siblings. We chose these covariates since they are not experiential factors but are variables that display individual differences that could contribute to vocabulary development in the HL (Paradis, 2023). We conducted a 1:1 nearest neighbor propensity score matching without replacement with a propensity score estimated using logistic regression of the treatment on the covariates. After matching, all standardized mean differences for the covariates were below 0.19 and all standardized mean differences for squares and two‐way interactions between covariates were below 0.05, indicating adequate balance as in Table 1.

**TABLE 1:** Summary of balance for matched data.

	Mean mono	Mean HSs	Std.Mean diff	Var.Ratio	eCDF mean	eCDF max	Std.Pair. dist
Distance	0.41	0.35	0.26	1.61	0.04	0.22	0.26
Age	6.69	7.24	−0.19	1.06	0.04	0.19	0.47
SES	4.55	4.46	−0.009	0.52	0.04	0.10	1.01
Gender	1.47	1.50	−0.08	1.00	0.02	0.04	0.82
Siblings	1.36	1.25	0.11	0.92	0.03	0.05	1.07

Sample sizes	HSs	Mono
All	423	136
Matched	136	136
Unmatched	287	0
Discarded	0	0

Abbreviations: eCDF, empirical cumulative distribution functions; HS, heritage speaker; SES, socioeconomic status.

**FIGURE 2:**
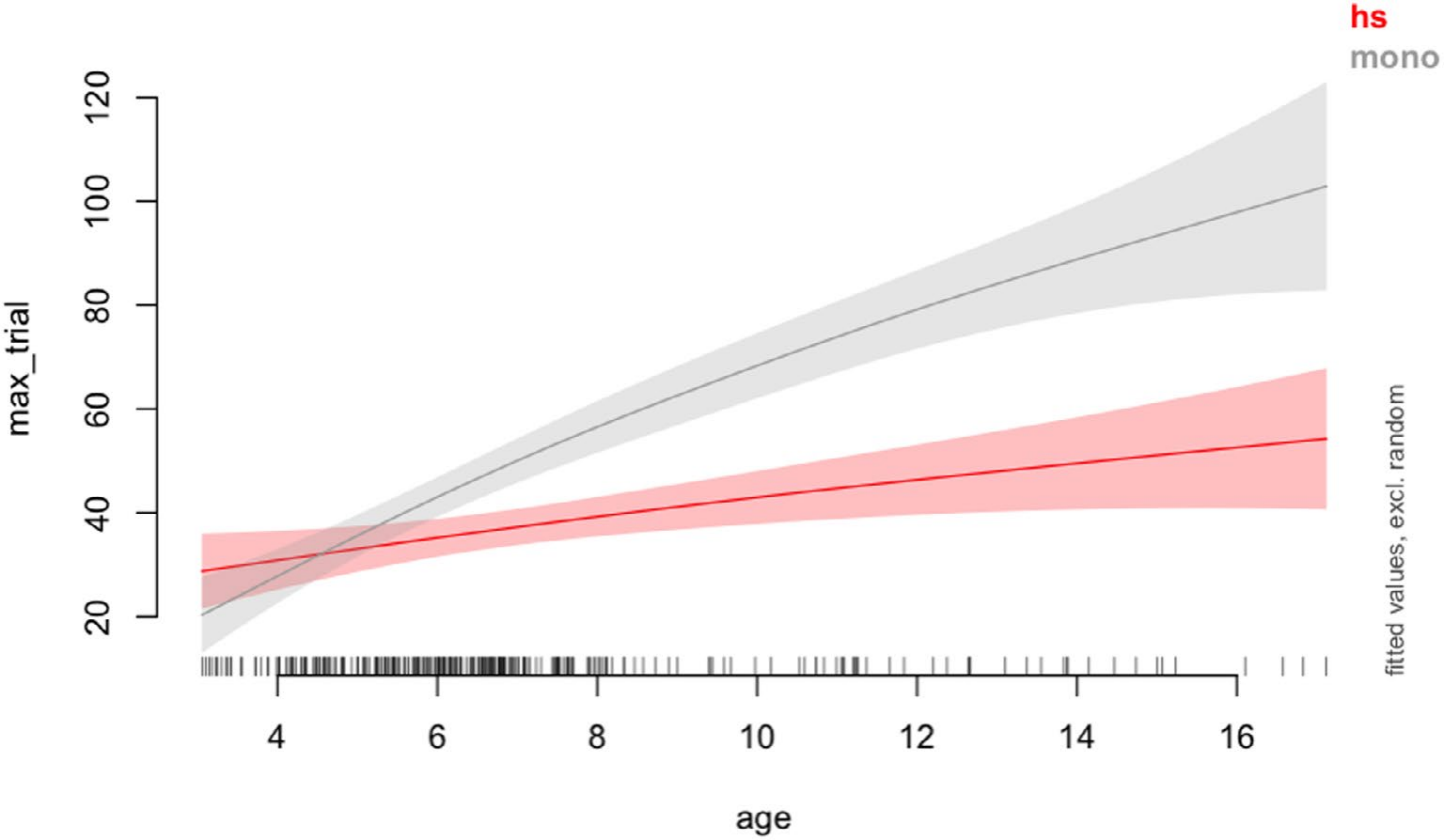
The interaction between age and group. Heritage speakers are indicated in red and monolinguals in gray. Shaded areas indicate 95% confidence intervals.

Upon matching the two groups in sample size and covariates, we ran a generalized additive mixed modeling (GAMM) using the “mgcv” package in R (Wood, 2011) to estimate when an effect of interest (i.e., significant difference in vocabulary scores between monolinguals and HSs) occurred and how long it lasted. GAMM can model both linear and nonlinear relationships and allows inclusion of random effects and slopes. However, since there is a single vocabulary score for each child, we fitted a GAMM without random effects for participant and item and included group and age (with smooth function to model nonlinear curves). The group variable was treatment coded and “monolinguals” was the reference level. The summary of the output is presented in Table 2. The output as well as the plot presented in Figure 2 using the “itsadug” package in R (Van Rij et al., 2015) shows that monolinguals have higher vocabulary scores than HSs and there are differences in the slope trajectory between the two groups. However, it is important to note here that the slopes are positive for both groups, but the monolinguals show a steeper positive slope than that of the HSs.

**TABLE 2:** Summary of the generalized additive mixed modeling.

	Estimate	SE	*t*	*p*
Parametric coefficients				
Intercept	36.88	1.69	21.70	<.001
Groupmono	11.41	2.40	4.74	<.001

	edf	Ref.df	*F*	*p*
Smooth terms				
s(age):grouphs	1.22	1.45	7.25	<.001
s(age):groupmono	1.87	2.34	48.76	<.001
*R* ^2^ = .33, deviance explained = 34.3%				

**FIGURE 3:**
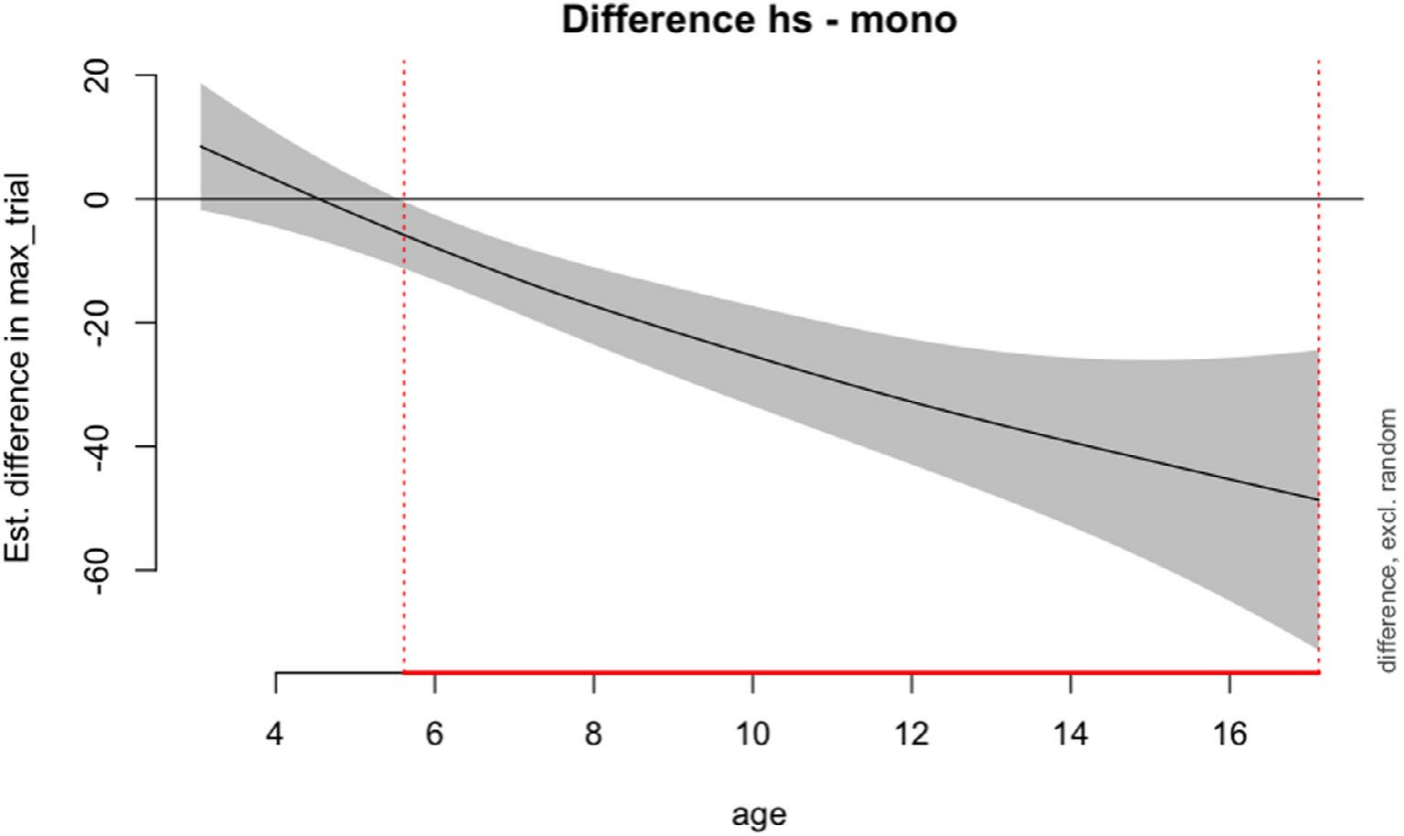
Vocabulary score difference plot for heritage speaker and monolingual groups. The shaded area indicates 95% confidence intervals. The red lines at the bottom indicate the age in which the difference in vocabulary scores between the two groups was significant.

The difference curves in Figure 3 plots the difference in vocabulary scores between the HSs and the monolinguals (HS—monolinguals) across the age span. The results show that significant differences in HS and monolinguals start to emerge at age 5.61, persisting through the oldest age available in this dataset—17.12 years.

### What are the underlying latent factors that can be extracted from the Q‐bex questionnaire and what bilingual constructs predict children's vocabulary knowledge in their HL?

Responses to the Q‐bex were examined with EFA, then evaluated with SEM in which the measurement model consists of fitting a confirmatory factor analysis (CFA). We first randomly split the data into two and ran an EFA with the first dataset. We included questions in the Q‐bex data that were related to the HL (Japanese) and not their societal language (English or German). These questions are listed in Table 3.

**TABLE 3:** List of questions from quantifying bilingual experience that are used for exploratory factor analysis.

How much the child hears/speaks Japanese in different contexts
Scale: 0–100
HL_ caregiver _to_child: At home, how often does the main caregiver use Japanese when speaking to the child? HL_child_to_ caregiver: At home, how often does child use Japanese when speaking to the main caregiver? HL_teacher_to_child: At school/day care, how often do teachers/carers use Japanese when speaking to the child? HL_child_to_teacher: At school/day care, how often does the child use Japanese when speaking to the teachers/carers? HL_friends_to_child: At school/day care, how often do friends use each language when speaking to the child? HL_child_to_friends: At school/day care, how often does the child use Japanese when speaking to friends? HL_friends_to_child_community: When the child is with friends in the local community (not at school/day care and not at home), how often do these friends use Japanese when speaking to the child? HL_child_to_friends_community: When the child is with friends in the local community (not at school/day care and not at home), how often does she or he use Japanese when speaking to them? HL_adults_to_child_community: When the child is with adults in the local community (not at school/day care and not at home), how often do these adults use Japanese when speaking to the child? HL_child_to_adults_community: When the child is with adults in the local community (not at school/day care and not at home), how often does she or he use Japanese when speaking to them? HL_adults_to_child_holiday: When the child was with adults during the school/day‐care holidays, how often did these adults use Japanese when speaking to the child? HL_child_to_adults_holiday: When the child was with adults during the school/day‐care holidays, how often did the child use Japanese when speaking to them? HL_children_to_child_holiday: When the child was with other children (including siblings) during the school/day‐care holidays, how often did these children use Japanese when speaking to the child? HL_child_to_children_holiday: When the child was with other children (including siblings) during the school/day‐care holidays, how often did the child use Japanese when speaking to them? HL_overheardspeech: How often do adults in the home speak in Japanese with other people in front of the child, but not directly to the child?

How frequent the child engages in different activities in Japanese
Scale: 0–4
freq_reading_HL: How often does the child read or is the child read to in Japanese? freq_writing_HL: How often does the child write in Japanese? freq_homework_HL: How often does the child do homework in Japanese? freq_schoollessons_HL: How often does the child have lessons about Japanese in regular school/day care (which include reading/writing)? freq_outschoollessons_HL: How often does the child have lessons about Japanese outside regular school/day care (which include reading/writing)? freq_tech_HL: How often does the child do any computer/technology‐related activities in Japanese? Consider activities such as TV, radio, music, films, websites, games, tablets, laptops, computers, phones, and apps. freq_timewfriends_HL: How often does the child interact with friends inside or outside the house in Japanese? freq_activites_outschool_HL: How often does the child participate in any activities outside regular school/day care in Japanese? Consider activities such as religious practice, sports, music, and cultural activities.

Family/internal information
Scale: 0–5 for number_of_siblings, 0–4 for HL_high_prof_speakers and HL_SES_caregiver1, 0–78 (months) for HL_onset and SL_onset
HL_SES_caregiver: Select the highest education level that caregiver completed in Japanese. number_of_siblings: How many siblings does the child have? nb_adults: How many adults does the child live with? HL_high_prof_speakers: How many people speak in Japanese to the child on a regular basis (i.e., at least once a week)? Consider: caregivers, siblings, other members of the household, family outside the household, friends/playmates, teachers, other significant people in child's life. HL_onset: age of onset to HL SL_onset: age on onset to the majority language (German or English)

Japanese proficiency
Scale: 0–4
HL_speaking: How well can your child speak Japanese for his or her age? HL_understanding: How well can your child understand Japanese for his or her age? HL_reading: How well can your child read in Japanese for his or her age? HL_writing: How well can your child write in Japanese for his or her age?

Abbreviations: HL, heritage language; SES, socioeconomic status; SL, Societal Language.

From an initial sample of 212 participants (half of the entire HSs randomly split into two groups), we deleted participants who had no responses in some of the questions, resulting in a final sample of 204 participants for the EFA. All responses were centered and scaled. As a first step in running an EFA, we ran a Kaiser–Meyer–Olkin test (a measure of how suited the data are for factor analysis) which showed that only three variables (i.e., number_of_siblings, HL_onset, and nb_adults) were below the value of 0.6, and thus these items were omitted from further analysis. Thirty items were analyzed with an ordinary least squares minimum residual approach to EFA using an oblique rotation (promax), allowing for factors to correlate. The eigenvalue method (i.e., Kairser's rule) suggests three factors to be extracted, while the scree plot suggested around six to seven, and the parallel plot six. Thus, we decided to obtain six factors in the subsequent analysis. Table 4 shows the factor loadings after rotation for the final analysis. Factor 1 represents “Holiday”: the amount of HL exposure and use between the child and other children as well as adults during stays in the homeland country (Japan) as well as amount of HL nochild‐directed speech (i.e., overheard speech). Factor 2 represents “School”: the amount of HL exposure and use between the child and other children or friends as well as teachers or caregivers during school in the ML environment. Factor 3 represents “Community”: the amount of HL exposure and use between the child and other children as well as adults in the local community (not at school or day care and not at home). Factor 4 represents “Proficiency”: self‐rated proficiency of HL speaking, understanding, reading, and writing. Factor 5 represents “Literacy”: how often the child is engaged in literacy activities in the HL such as reading, writing, and homework. Factor 6 represents “Home”: the amount of HL exposure and use between the child and the main caretaker as well as the final education of the main caretaker in the HL. These six factors accounted for 61.80% of the total variance.

**TABLE 4:** Factor loadings (pattern matrix) from the factor analysis.

	F1: Holiday	F2: School	F3: Community	F4: Prof	F5: Literacy	F6: Home
HL_caregiver _to_child	−0.08	0.00	0.01	0.05	−0.05	**1.02**
HL_child_to_caregiver	0.10	−0.02	−0.01	0.28	−0.04	**0.73**
HL_teacher_to_child	−0.02	**1.00**	−0.09	−0.08	−0.01	−0.03
HL_child_to_teacher	−0.01	**1.00**	−0.08	−0.08	−0.00	−0.01
HL_friends_to_child	−0.06	**0.95**	0.06	−0.02	−0.01	0.03
HL_child_to_friends	−0.05	**0.90**	0.05	0.00	0.00	0.03
HL_friends_to_child_community	−0.10	−0.02	**0.91**	0.09	−0.04	−0.02
HL_child_to_friends_community	−0.13	0.10	**0.91**	0.11	−0.09	0.01
HL_adults_to_child_community	0.11	−0.13	**0.93**	−0.12	0.05	−0.03
HL_child_to_adults_community	0.10	−0.02	**0.93**	−0.10	−0.01	−0.00
HL_adults_to_child_holiday	**0.95**	−0.04	−0.09	0.04	0.05	−0.04
HL_child_to_adults_holiday	**0.95**	−0.01	−0.12	0.14	−0.03	−0.01
HL_children_to_child_holiday	**0.76**	0.05	0.07	0.23	−0.12	−0.03
HL_child_to_children_holiday	**0.76**	0.05	0.07	0.23	−0.12	−0.04
HL_overheardspeech	**0.56**	−0.04	0.06	−0.05	0.13	0.01
HL_high_prof_speakers	0.15	0.01	0.00	0.20	−0.08	0.01
freq_reading_HL	0.01	0.10	−0.04	0.37	0.26	0.02
freq_writing_HL	0.10	0.02	−0.00	0.05	**0.85**	−0.03
freq_homework_HL	0.15	0.06	−0.05	−0.15	**0.91**	−0.02
freq_schoollessons_HL	0.11	0.07	0.08	−0.01	**0.42**	−0.01
freq_outschoollessons_HL	−0.03	0.01	−0.05	−0.06	**0.51**	−0.01
freq_tech_HL	0.16	0.02	−0.01	0.43	0.04	0.00
freq_timewfriends_HL	0.03	0.09	0.19	0.14	0.14	0.02
freq_activites_outschool_HL	−0.09	−0.06	0.02	0.01	0.28	0.04
HL_SES_caregiver	0.03	0.02	−0.03	−0.19	0.05	**0.74**
HL_speaking	−0.06	−0.09	0.05	**0.88**	−0.07	−0.01
HL_understanding	−0.04	−0.02	0.00	**0.86**	−0.02	−0.02
HL_reading	−0.06	−0.09	−0.08	**0.57**	**0.41**	−0.05
HL_writing	−0.09	−0.08	−0.04	**0.48**	**0.44**	−0.01
SL_onset	**0.43**	−0.04	0.01	−0.20	0.03	0.04

*Note*: Factor loadings greater than .40 are in bold.

Abbreviations: HL, heritage language; SES, socioeconomic status.

We also ran a Cronbach's *α* reliability to make sure that the factors were appropriately grouped. The *α* values of all common factors ranged between .70 and .96, and were all greater than the required minimum of .60 (DeVellis & Thorpe, 2021), indicating that individual dimension possessed good internal consistency.

In order to examine how the factors extracted from EFA predict children's vocabulary knowledge, we employed SEM which is a statistical method used to analyze the relationships between observed and latent variables. SEM uses a combination of CFA and regression analysis to test a theoretical model of the relationships between variables. The model is represented graphically as a path diagram, where the arrows indicate the direction of the relationship between variables. The model is then tested using statistical methods to determine how well it fits the observed data.

We ran a SEM with the remaining half of the data which initially consisted of 215 participants, but exclusion of missing data resulted in a total of 207 participants for the final second half of the dataset. The SEM was fitted using the lavaan package (Rosseel, 2012) in R. In the preceding analysis, dimension reduction and item classification of the data by EFA provided a foundation for understanding the causal relationships to construct the measurement and structural models of SEM. Thus, an item with a factor loading of more than 0.40 in the EFA (bolded items in Table 4) was included in the measurement model. To evaluate the interactions among the extracted common factors, the following hypotheses were developed:

- Five latent factors related to HL engagement (Holiday, School, Community, Literacy, Home) should predict vocabulary scores.
- Five latent factors related to HL engagement (Holiday, School, Community, Literacy, Home) should predict (self‐ or parent‐reported) Proficiency latent factor.
- Vocabulary scores and Proficiency latent factor should correlate.

**FIGURE 4:**
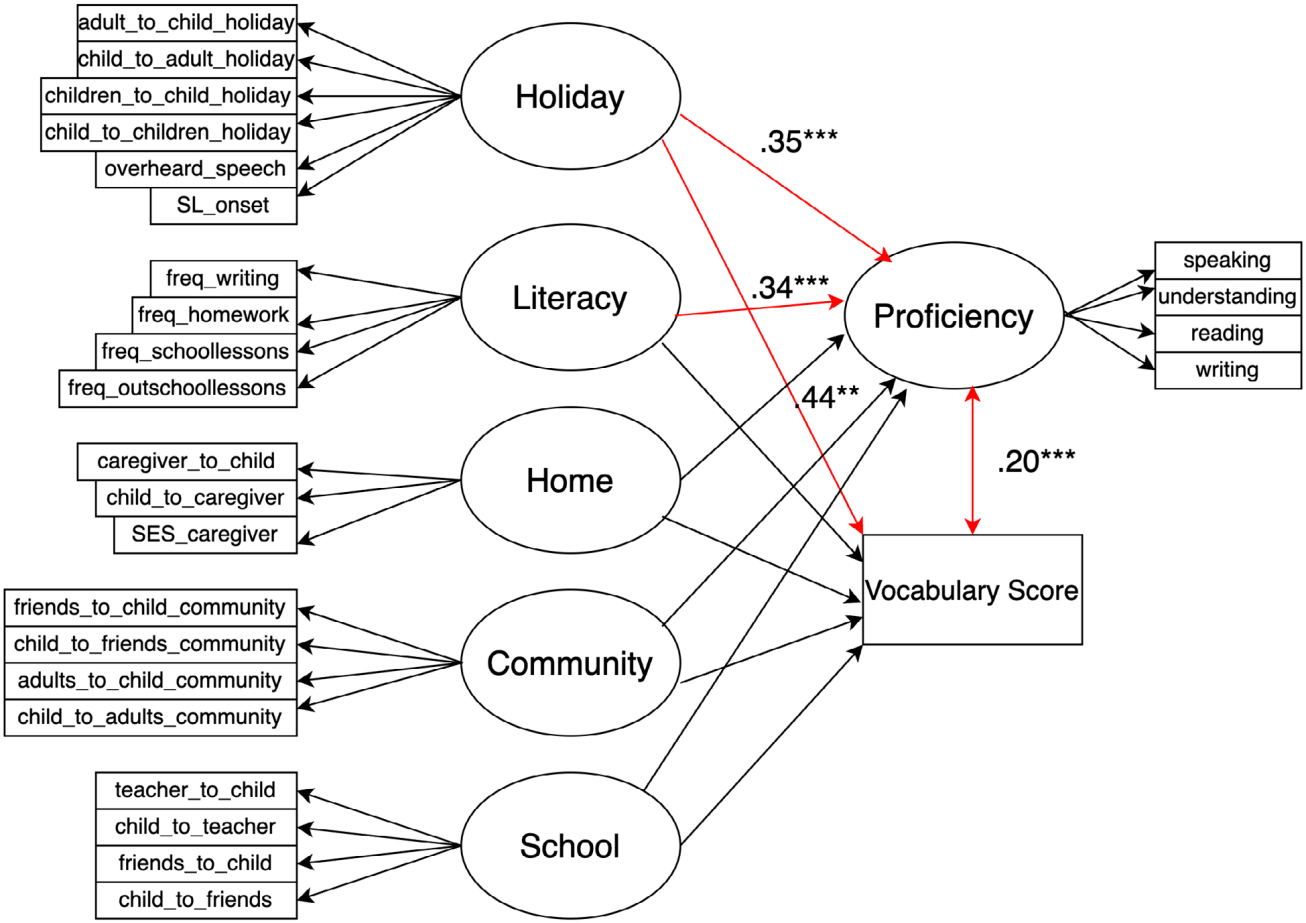
Structural equation modeling plot and path estimates. **p < .01, and ***p < .001. Significant regression path coefficients are in red.

Based on the earlier hypothesis (used for building the structural model) and with the measurement model established, a SEM containing both the measurement model and the structural model was then developed as illustrated in Figure 4. We also included the following residual covariances in the model which include: HL_friends_to_child_community and HL_child_to_friends_community, HL_adults_to_child_community and HL_child_to_adults_community, HL_teacher_to_child and HL_child_to_teacher, HL_friends_to_child and HL_child_to_friends, HL_adults_to_child_holiday and HL_child_to_adults_holiday, HL_children_to_child_holiday and HL_child_to_children_holiday, HL_speaking and HL_understanding, and HL_reading and HL_writing. These residual covariances were included as it was expected that a child's exposure and use in a certain context will correlate. Furthermore, speaking and understanding as well as reading and writing skills were also expected to correlate. The full model specification can be found via the R file in https://osf.io/ukyg4/.

The model summary (see Supporting Information for the full output) indicates an acceptable fit of the model (Kline, 2011) with root mean square error of approximation less than .08 (.06), comparative fit index higher than .90 (.95), Tucker–Lewis index higher than .90 (.94), and standardized root mean square residual less than .08 (.05). The only indices that did not meet the requirement was the chi‐square t test, which was highly significant (<.001). However, since chi‐square tests of exact fit have often been found to reject the null hypothesis, especially in large samples (Shi et al., 2019), and because all other indices complied with the operational standards, the optimized model was deemed acceptable. The output indicated that all observation variables in the measurement model loaded onto each factor significantly (*p*s < .001).

Table 5 outlines the path coefficients of the five latent variables on observed vocabulary score and Proficiency latent variable. The results here indicate that Holiday significantly predicted vocabulary score and Holiday and Literacy significantly predicted (self‐ or parent‐rated) Proficiency. There was also a significant correlation between vocabulary score and Proficiency (*E* = .20, *p* < .001).

**TABLE 5:** Path coefficients of the latent variables in the optimized structural equation model.

Relationship	Path coefficient	*p*
Community → vocabulary score	.02	.84
School → vocabulary score	.03	.72
Holiday → vocabulary score	.44	.001
Literacy → vocabulary score	−.03	.71
Home → vocabulary score	−.002	.95
Community → proficiency	.13	.11
School → proficiency	−.05	.40
Holiday → proficiency	.35	<.001
Literacy → proficiency	.34	<.001
Home → proficiency	.02	.47

## DISCUSSION

This study examined the receptive vocabulary of Japanese monolingual and HS children to identify when their vocabulary knowledge starts to diverge and what factors may account for individual differences. By carefully matching the two groups on environment or internal related factors such as age, SES, number of siblings, and gender, we were able to isolate the effects of age on vocabulary development in these two groups of L1 speakers of Japanese. The results show that between the ages of 4 and 5, there are no significant differences in the vocabulary score between the monolinguals and HSs. However, at age 5.61 years (around 5 years and 7 months old), HSs begin to diverge from their monolingual peers (with HSs scoring lower) and this difference continuously persisting until they reach young adulthood of 17.12 years.

Much previous research has suggested that ages 5 to 6 is a crucial time window in which the dominance of language exposure between the HL and the ML starts to shift due to onset of schooling, which is characterized by an increase in exposure and use to the ML and consequently a decrease in exposure to and use of the HL (Bialystok et al., 2010; Dubiel & Guilfoyle, 2017; Gathercole & Thomas, 2009; Ortega, 2020; Rodina et al., 2023; Sheng et al., 2011). Indeed, the majority of the HSs in this study lived in the United States or Germany—both countries in which compulsory education begins around age 5 to 6 (in the United States, age of required school attendance differs across states).

Our results are in line with Rodina et al. (2023), which examined the lexical development of 143 Russian HS children in Norway, Germany, and the United Kingdom via a narrative task. Their findings showed that the number of different words in the ML became greater than those of HL around age 5 for children from families with two Russian‐speaking parents, suggesting that shift in language dominance at the lexical level interacts with the amount of language exposure children receive in the home. Although Rodina et al.'s study compared the dominance of lexical productive abilities between the two languages of the HSs, it is not surprising that the present data (a comparison between HSs to monolingual counterparts in the HL) converge with theirs, as both patterns of divergence are most likely a consequence of a dominance shift in quantitative and qualitative language exposure and use. In a similar line of work, Thordardottir (2011) examined receptive and expressive vocabulary knowledge among 84 French–English bilingual children, ages 4 to 5, with various amounts of exposure in the two languages while controlling for age, SES, and nonverbal intelligence. Their findings show that for receptive vocabulary, children who had received around half of their exposure in the target language (or more) performed similarly to their monolingual peers (both in terms of English and French), while for expressive vocabulary, more than 60% of exposure was needed to achieve performance comparable to that of the monolingual group. Their findings provide a complementary perspective to our results by demonstrating that the HL vocabulary—on a group level—begins to diverge from monolinguals shortly after entering the school system in the ML, but this is not the case for *all* HSs and thus, maintaining a certain level of threshold in HL exposure is crucial to fill this gap.

It is not surprising that exposure is an important factor in bilingual children's vocabulary development, especially in the HL where the amount and the quality of exposure varies greatly from individual to individual. However, it is yet unclear what aspects or contexts of exposure or use are more or less responsible for HL development. Paradis (2023) identifies several internal and external factors that account for individual variation in the HL, which include (a) internal factors such as age at L2 acquisition, cognitive abilities, socioemotional well‐being, (b) proximal factors such as cumulative exposure to HL, HL use at home, and richness of HL environment, and (c) distal factors such as literacy and education in HL, parent proficiency in HL, SES, and family attitudes or identities. In addition to the fact that there is great variability in documenting such facets of bilingualism due to differences in the questionnaires used and operationalization of particular components of bilingual experience (see Kašćelan et al., 2022 for further discussions), most studies employ an extensive questionnaire but only use responses from a single question to directly predict HL abilities (unless they use composite or aggregate scores as provided by questionnaires such as Language and Social Background Questionnaire, Anderson et al., 2018; Language History Questionnaire, Li et al., 2020). For instance, Sun et al. (2020) emphasizes the importance of the family's participation in general language and literacy activities on vocabulary development, as they found that the number of books in the HL significantly predicted children's receptive vocabulary size. However, the number of HL books at home was measured via a broad Likert scale (1 = none, 2 = 1–10 books, 3 = 11–30 books, 4 = 31–60 books, 5 = 61–90 books, 6 = 91–120 books, 7 = more), and thus, such crudely observed values may not accurately reflect their intended underlying construct (i.e., literacy activities) due to various sources of noise, bias, or imprecision in the measurement process.

By using a fit‐for‐purpose questionnaire for heritage bilingualism (Q‐bex, De Cat, 2023) that covers most of the bilingual dimensions identified by Paradis (2023), and using EFA to uncover the underlying structure of the observed variables without preconceived notions regarding how they should group together, we were able to identify six factors that characterize the HSs in our sample. These included (1) “Holiday”: the amount of HL exposure and use between the child and other children as well as adults during stays in the homeland country as well as amount of HL non‐child‐directed speech (i.e., overheard speech), (2) “School”: the amount of HL exposure and use between the child and other children or friends as well as teachers or carers during school in the ML environment, (3) “Community”: the amount of HL exposure and use between the child and other children as well as adults in the local community (not at school or day care and not at home), (4) “Proficiency”: self‐rated proficiency of HL speaking, understanding, reading, and writing, (5) “Literacy”: how often the child is engaged in literacy activities in the HL such as reading, writing, and homework, (6) “Home”: the amount of HL exposure and use between the child and the main caretaker as well as the final education of the main caretaker in the HL.

Since Q‐bex was a newly established questionnaire, the initial exploration via EFA helped us to understand the complexity of the data and how well the observed variables align with hypothesized latent constructs. We then built a SEM, including the measurement model that evaluates how well a predefined factor structure (based on the EFA) fits the observed data. By comparing the factor structure obtained from EFA to the model specified in SEM, we were able to confirm that the stability and consistency of the latent structure was robust across different datasets (randomly split into two).

We found from the structural modeling of SEM that Holiday predicted both vocabulary scores and (self‐rated) Proficiency, while Literacy predicted (self‐ or parent‐rated) Proficiency. The crucial role of Literacy for vocabulary development supports findings of several previous studies that had also found a significant effect of literacy engagement on vocabulary such as the number of books (Rydland & Grøver, 2021; Sun et al., 2020, 2022), frequency of reading to the child and frequency of maternal questions during book sharing (Quiroz et al., 2010), school‐work–related reading practice (Zhang & Koda, 2011), print and media exposure (Ryan, 2021), and how often the parents read to themselves and how often they read storybooks to their children (Willard et al., 2015). Our findings also complement the results of Mori and Calder (2017) who found that frequency of reading for pleasure predicted the HL vocabulary of older Japanese HSs in the United States (ages 15–18).

It is also not surprising that Community and School did not predict either self‐rated proficiency or vocabulary scores, since the average HL exposure for questions related to Community was less than 22% and the average HL exposure for questions related to School was less than 8%. Thus, these predictors may have lacked adequate variation in order to estimate reliable coefficients and accurately assess the model's performance to make meaningful predictions in the present dataset. This is probably due to the fact that Japanese is not commonly taught as a foreign or second language globally, except for in neighboring countries such as China, Korea, and Indonesia (see Japan Foundation, 2021 for detailed report) and a large Japanese diaspora exists only in very limited areas of the countries in which the current participants grew up, such as Los Angeles, Honolulu, Sydney, London, and Düsseldorf.

A novel aspect of our findings is that HL exposure and use during the holidays as well as overheard speech predicted both self‐rated proficiency and vocabulary scores. We interpret this factor as a proxy for immersion experience by temporarily returning to their (parents') country of origin (in this case Japan). Thus, the higher the factor scores are for Holiday, the more opportunities the children will have had to engage with Japanese with increased input diversity and dispersion in a context where it potentially is the only viable linguistic option. Indeed, increased diversity in exposure to sources of input (i.e., from different speakers and contexts) has been shown to be nontrivial not only for HSs (Gollan et al., 2015), but also for monolingual (Huttenlocher et al., 2010; Rowe, 2012) and L2 speakers (Barcroft & Sommers, 2005; Sinkeviciute et al., 2019). Visiting the heritage homeland also means that Japanese HSs will find themselves having increased opportunities for (enforced) engagement with HL production. Although this might differ depending on the HL and its specific homeland context, in societies like Japan that are truly functionally monolingual, communication with family members and friends and existing in the general society (as a nontourist) will entail a need to speak the HL (Japanese). As a consequence, such HSs may encounter words that they are typically not exposed to in their country of residence, or have the chance to reactivate words that are infrequent in the input (Chondrogianni & Daskalaki, 2023). Moreover, from a sociocultural perspective, studies have shown even short visits to the homeland to be beneficial in strengthening emotional ties to the homeland culture and establishing a stronger ethnic identity (Petrucci, 2007).

To our knowledge, Chondrogianni and Daskalaki (2023) is the only study that has specifically examined the effect of homeland visits during holiday on HL vocabulary development in children. They show that visits to the country of origin (measured in weeks cumulatively over the past 4 years) was a stronger predictor than current HL use for HS's expressive vocabulary. Our results also show no influence of Home on vocabulary development, but a robust positive effect of Holiday on both self‐ or parent‐reported Proficiency and vocabulary scores. Our findings add new insights into this line of work by going beyond their simple operationalization of frequency of homeland visits (measured in weeks) by using latent factors that incorporate quantity (how frequent) and quality (with whom) of interactions in HL during the holidays. It is expected that, in addition to literacy engagement, HL activities such as immersion experience that goes “above and beyond” the exposure that HSs normally receive may be especially important for vocabulary knowledge, a vulnerable domain given its obvious sensitivity to input.

The importance of immersion to the L1 environment on HL development has also been demonstrated in a specific group of bilingual children—namely returnees, who are children of immigrant families who spend a significant portion of their formative developmental years in a foreign ML context, a typical HL scenario, yet return to their native HL environment, often as older children or teenagers (Flores & Kubota, 2023). Although limited, studies have shown that returnee children, even after a short period of reimmersion to the homeland, rapidly improve their (former) HL, especially at the lexical level (Flores et al., 2022; Kubota et al., 2020, 2022). For instance, Kubota et al. (2020) showed faster lexical access in Japanese after a year of reimmersion in Japan, and Kubota et al. (2022) demonstrated that children who were more dominant in the ML (English) were able to catch up to other returnees who were either more dominant in Japanese or balanced in English and Japanese in terms of lexical diversity (Type–Token ratio) after only a year in the homeland. Perhaps quantity and quality of immersion experience in the homeland may have been overlooked as a potential predictor in previous work on HLB, due to the fact that it only comprises a small portion of any given child's time (typically at a maximum 1 to 2 months per year). Furthermore, it does not pertain to all HSs (not all families are in the position to make such trips), and the frequency of visits can vary over time, thus making it either something difficult to measure or simply deemed as less important than other factors such as language exposure and use at home. We recommend future work to include questions related to homeland visits when documenting the language experiences of HSs, as it has explanatory use of observed HL variances—at least under certain conditions such as the ones that characterize Japanese in our present participants—more than one might expect.

Finally, the SEM model showed that Proficiency latent factor (extracted via self‐ or parent‐reported Proficiency) correlated significantly with vocabulary scores. This was to be expected, as many other studies have found a positive relationship between self‐ or parent‐reported proficiency and vocabulary knowledge (Lemhöfer & Broersma, 2012; Luk & Bialystok, 2013; Tomoschuk et al., 2019), indicating that measures of subjective and objective proficiency can indeed capture some common variances. However, one should be cautious when choosing which measures to use as a proxy for “Proficiency” as a dependent variable, as we show in our findings that different (and shared) bilingual factors regress onto self‐ or parent‐reported measures and vocabulary scores. We speculate that literacy was a better predictor for self‐ or parent‐reported Proficiency than vocabulary scores, since those that read and write more frequently and attend supplementary Saturday schools may have had a more holistic perception of their *Proficiency*, beyond the aspect of vocabulary (such as syntax, semantics, and pragmatics). This ties into the discussion of the operationalization of *Proficiency* and what the pros and cons are in terms of using an objective measure like vocabulary scores which measure only one aspect (lexicon) of the linguistic knowledge, or a subjective measure like self‐rated Proficiency that can be prone to measurement errors but may provide a more holistic representation of one's linguistic knowledge. Although these two measures can both be defined as a single construct—*Proficiency*—and are often used interchangeably in empirical work, the choices we make in terms of its operationalization can have major consequences for our analyses and findings.

### Limitations and future directions

Although the sample in our study was fairly large and covered a wide age range of HSs, the study was cross‐sectional in nature. Given our approach, we were not able to track the individual development and change or maintenance of HL over time in childhood, despite acknowledging that such longitudinal data would be important to test the validity of the present interpretations. Indeed, in order to uncover the complex and dynamic nature of HL development and to truly hone in on individual differences in developmental trajectories, the field needs longitudinal studies that span over several years to capture changes in the same individuals over time to best model how internal, proximal, and distal factors interact and affect the development of HL (Montrul, 2018). However, to understand not just the point of divergence from monolinguals—a question of limited value beyond its descriptive nature—but indeed the trajectory of continued development and at the individual HS level, a longitudinal version of this study would still require 10 years of tracking. Of course, conducting on scale such a study due to its financial resource and labor needs, not to mention complexities with participant and staff attrition over such an extended period, is not to be taken lightly. Therefore, we recommend future work to carry out studies that combines cross‐sectional and longitudinal methodologies, by, for example, testing a group of 5‐, 8‐, 11‐, and 14‐year‐olds over the course of 3 years. This will allow us to examine in both real time (because each participant is tested over a 3‐year span of personal development) and apparent time (because we combine four cohorts of increasing ages year by year from 5 to 16) the crucial time frame for when changes in HL are most likely to obtain.

Despite the fact that our large‐scale study hints at some generalizable implications for educational practice and policy, we certainly do not want to make claims that literacy and immersion experience during the holidays are deterministic for vocabulary development in (Japanese) HSs. This is, first, because predictive factors for HL vocabulary may differ in other language‐dominant countries that is more typologically similar to Japanese such as Korea (i.e., more cognates between Japanese and Korean than Japanese and English or German), or countries that are geologically closer to Japan such as China, Singapore, and Malaysia (e.g., it is easier to return to Japan for holidays than from North America or Europe). Second, the parents' capacity to provide such environments and opportunities for their children may differ substantially depending on the parents' literacy levels, SES, income, cultural beliefs and practices, family structure, and the resources available in the community (Paradis, 2023). Indeed, returning to Japan on a frequent basis comes with a potentially differential, relative financial cost on specific families and literacy engagement may be extremely difficult for those who live in a community where there are no supplementary Saturday schools (*hoshuuko*). Despite such concerns, perhaps we can offer more general recommendations by encouraging heritage bilingual families to broaden their social networks wherever they find themselves and create as many contexts in which their child can interact with a maximum amount of speakers of the HL—for example (but not limited to), by visiting the home country, participating in exchange programs, talking to families and friends via the Internet, having babysitters that are L1 speakers of the HL, and creating playgroups (or an age‐appropriate counterpart such a reading club for teens) where peer‐to‐peer interactions in the HL are facilitated, valued, and encouraged.

Finally, although children were informed that they can quit the task anytime and their participation is voluntary, we only asked for explicit consent from the parents, and did not ask for the child's consent. Obtaining child consent in experiments and studies is crucial for ethical and legal reasons, ensuring that the child's autonomy and rights are respected (Graham et al., 2014). Informed assent involves clearly explaining the study's purpose, procedures, and potential risks and benefits in an age‐appropriate manner, allowing the child to make an informed decision about participation. Child consent, alongside parental consent, ensures that children are aware of their participation and its implications, which can reduce anxiety and improve the validity of the study. Additionally, it aligns with ethical guidelines and regulations that safeguard the well‐being of child participants in research (Christensen & James, 2017). Thus, we strongly recommend future work to obtain child consent in addition to parental consent, whether or not required by internal ethical approval bodies, especially when administering a task online. In particular, the right to withdraw from the study at any time without penalty is crucial, and researchers should reiterate this right throughout the study, ensuring children feel comfortable and secure in their participation decisions. These considerations help build trust, protect children's rights, and enhance the ethical integrity of the research.

## CONCLUSION

This study examined when the receptive vocabulary knowledge of HSs begin to diverge from their monolingual peers and what factors explain their developmental trajectory. We matched the monolinguals and HSs on various covariate factors such as age, gender, and SES so that we could compare their vocabulary scores from young childhood to adolescence. We found that HSs performed similarly to the monolinguals until 5.61 years old and beyond this point, the difference in vocabulary scores persisted until the latest age of testing (17.12 years old). In terms of the factors that predicted vocabulary development in the present HSs, we identified six latent factors (Holiday, School, Community, Proficiency, Literacy, and Home). The EFA and subsequent SEM regression paths indicated that Holiday predicted vocabulary score and Literacy and Holiday predicted (self‐ or parent‐rated) Proficiency. Our findings underscore the significance of early immersion experiences during holidays and the role of literacy engagement in HL development, while also emphasizing the intricate interplay of diverse background factors in shaping different aspects of proficiency.

## Supporting information


Data S1.

## Data Availability

The data necessary to reproduce the analyses presented here are publicly accessible. Data are available at the following URL: https://osf.io/ukyg4/. The analytic code necessary to reproduce the analyses presented in this article is publicly accessible. Code is available at the following URL: https://osf.io/ukyg4/. The materials necessary to attempt to replicate the findings presented here are publicly accessible: https://app.gorilla.sc/openmaterials/686845. The analyses presented here were not preregistered.
