# Supplementary material for: Modeling individual differences in vocabulary development: A large‐scale study on Japanese heritage speakers
Source: Child Dev. 2024 Sep 29;96(1):325–40. doi: 10.1111/cdev.14168 (PMC11693831; doi:10.1111/cdev.14168)
Supplement: Supplementary file 1 — Data S1. [file CDEV-96-325-s001.docx]

**Supplementary Materials**

S.1.Full model output of Structural Equation Modelling

lavaan 0.6-11 ended normally after 99 iterations

Estimator ML

Optimization method NLMINB

Number of model parameters 81

Number of observations 207

Model Test User Model:

Test statistic 530.213

Degrees of freedom 270

P-value (Chi-square) 0.000

Model Test Baseline Model:

Test statistic 5509.036

Degrees of freedom 325

P-value 0.000

User Model versus Baseline Model:

Comparative Fit Index (CFI) 0.950

Tucker-Lewis Index (TLI) 0.940

Loglikelihood and Information Criteria:

Loglikelihood user model (H0) -5740.241

Loglikelihood unrestricted model (H1) -5475.135

Akaike (AIC) 11642.483

Bayesian (BIC) 11912.433

Sample-size adjusted Bayesian (BIC) 11655.789

Root Mean Square Error of Approximation:

RMSEA 0.068

90 Percent confidence interval - lower 0.060

90 Percent confidence interval - upper 0.077

P-value RMSEA <= 0.05 0.000

Standardized Root Mean Square Residual:

SRMR 0.058

Parameter Estimates:

Standard errors Standard

Information Expected

Information saturated (h1) model Structured

Latent Variables:

Estimate Std.Err z-value P(>|z|) Std.lv Std.all

Community =~

HL_frnds_t_ch_ 1.000 0.875 0.877

HL_chld_t_frn_ 1.032 0.024 43.913 0.000 0.903 0.905

HL_dlts_t_chl_ 0.956 0.080 11.963 0.000 0.836 0.838

HL_chld_t_dlt_ 1.005 0.079 12.689 0.000 0.879 0.881

School =~

HL_tchr_t_chld 1.000 0.871 0.873

HL_chld_t_tchr 1.021 0.027 38.066 0.000 0.889 0.891

HL_frnds_t_chl 1.088 0.097 11.198 0.000 0.948 0.950

HL_chld_t_frnd 1.105 0.097 11.342 0.000 0.963 0.965

Holiday =~

HL_dlts_t_chl_ 1.000 0.791 0.793

HL_chld_t_dlt_ 1.111 0.039 28.845 0.000 0.880 0.882

HL_chldrn_t_c_ 1.072 0.085 12.637 0.000 0.848 0.850

HL_chld_t_chl_ 1.103 0.084 13.096 0.000 0.873 0.875

HL_overhrdspch 0.648 0.089 7.322 0.000 0.513 0.514

SL_onset 9.834 1.678 5.862 0.000 7.783 0.418

Literacy =~

freq_writng_HL 1.000 0.824 0.826

freq_homwrk_HL 0.988 0.099 9.929 0.000 0.813 0.815

frq_tschlls_HL 0.210 0.092 2.286 0.022 0.173 0.174

frq_schllss_HL 0.695 0.090 7.745 0.000 0.573 0.574

Home =~

HL_dlt1_t_chld 1.000 1.237 1.240

HL_chld_t_dlt1 1.010 0.066 15.298 0.000 1.249 1.252

HL_SES_cargvr1 0.414 0.098 4.205 0.000 0.512 0.513

Proficiency =~

HL_speaking 1.000 0.718 0.720

HL_understndng 0.759 0.083 9.167 0.000 0.545 0.546

HL_reading 0.958 0.124 7.750 0.000 0.688 0.689

HL_writing 0.987 0.124 7.952 0.000 0.709 0.710

Regressions:

Estimate Std.Err z-value P(>|z|) Std.lv Std.all

max_trial ~

Community 0.022 0.114 0.196 0.845 0.020 0.020

School 0.031 0.089 0.354 0.723 0.027 0.027

Holiday 0.447 0.129 3.477 0.001 0.354 0.355

Literacy -0.035 0.095 -0.372 0.710 -0.029 -0.029

Home -0.002 0.036 -0.057 0.954 -0.003 -0.003

Proficiency ~

Community 0.136 0.087 1.565 0.118 0.165 0.165

School -0.056 0.067 -0.842 0.400 -0.068 -0.068

Holiday 0.358 0.100 3.587 0.000 0.395 0.395

Literacy 0.341 0.077 4.413 0.000 0.391 0.391

Home 0.020 0.028 0.714 0.475 0.035 0.035

Covariances:

Estimate Std.Err z-value P(>|z|) Std.lv Std.all

.HL_friends_to_child_community ~~

.HL_chld_t_frn_ 0.172 0.051 3.353 0.001 0.172 0.844

.HL_adults_to_child_community ~~

.HL_chld_t_dlt_ 0.212 0.050 4.204 0.000 0.212 0.824

.HL_adults_to_child_holiday ~~

.HL_chld_t_dlt_ 0.231 0.040 5.750 0.000 0.231 0.809

.HL_children_to_child_holiday ~~

.HL_chld_t_chl_ 0.222 0.038 5.784 0.000 0.222 0.874

.HL_teacher_to_child ~~

.HL_chld_t_tchr 0.168 0.063 2.680 0.007 0.168 0.766

.HL_friends_to_child ~~

.HL_chld_t_frnd 0.026 0.071 0.367 0.714 0.026 0.320

.HL_adult1_to_child ~~

.HL_chld_t_dlt1 -0.809 0.309 -2.620 0.009 -0.809 -1.475

.HL_speaking ~~

.HL_understndng 0.294 0.062 4.747 0.000 0.294 0.507

.HL_reading ~~

.HL_writing 0.259 0.062 4.177 0.000 0.259 0.511

Community ~~

School 0.327 0.067 4.858 0.000 0.430 0.430

Holiday 0.416 0.067 6.177 0.000 0.601 0.601

Literacy 0.177 0.061 2.900 0.004 0.245 0.245

Home 0.303 0.063 4.850 0.000 0.280 0.280

School ~~

Holiday 0.166 0.055 2.998 0.003 0.241 0.241

Literacy 0.204 0.061 3.354 0.001 0.284 0.284

Home 0.093 0.054 1.730 0.084 0.087 0.087

Holiday ~~

Literacy 0.175 0.056 3.118 0.002 0.268 0.268

Home 0.468 0.070 6.722 0.000 0.478 0.478

Literacy ~~

Home 0.100 0.055 1.826 0.068 0.098 0.098

.Proficiency ~~

.max_trial 0.203 0.050 4.045 0.000 0.405 0.436

Variances:

Estimate Std.Err z-value P(>|z|) Std.lv Std.all

.HL_frnds_t_ch_ 0.230 0.053 4.333 0.000 0.230 0.231

.HL_chld_t_frn_ 0.180 0.052 3.475 0.001 0.180 0.181

.HL_dlts_t_chl_ 0.296 0.053 5.557 0.000 0.296 0.298

.HL_chld_t_dlt_ 0.223 0.051 4.355 0.000 0.223 0.224

.HL_tchr_t_chld 0.236 0.063 3.733 0.000 0.236 0.237

.HL_chld_t_tchr 0.204 0.064 3.180 0.001 0.204 0.205

.HL_frnds_t_chl 0.096 0.071 1.354 0.176 0.096 0.097

.HL_chld_t_frnd 0.068 0.072 0.945 0.345 0.068 0.069

.HL_dlts_t_chl_ 0.369 0.049 7.570 0.000 0.369 0.371

.HL_chld_t_dlt_ 0.221 0.038 5.797 0.000 0.221 0.222

.HL_chldrn_t_c_ 0.276 0.041 6.672 0.000 0.276 0.277

.HL_chld_t_chl_ 0.233 0.038 6.088 0.000 0.233 0.235

.HL_overhrdspch 0.732 0.075 9.770 0.000 0.732 0.735

.SL_onset 286.596 28.840 9.937 0.000 286.596 0.826

.freq_writng_HL 0.317 0.063 5.024 0.000 0.317 0.318

.freq_homwrk_HL 0.334 0.063 5.309 0.000 0.334 0.335

.frq_tschlls_HL 0.965 0.095 10.110 0.000 0.965 0.970

.frq_schllss_HL 0.667 0.073 9.132 0.000 0.667 0.670

.HL_dlt1_t_chld -0.534 0.314 -1.702 0.089 -0.534 -0.537

.HL_chld_t_dlt1 -0.564 0.319 -1.769 0.077 -0.564 -0.567

.HL_SES_cargvr1 0.733 0.088 8.298 0.000 0.733 0.737

.HL_speaking 0.480 0.069 6.916 0.000 0.480 0.482

.HL_understndng 0.698 0.081 8.662 0.000 0.698 0.702

.HL_reading 0.522 0.073 7.136 0.000 0.522 0.525

.HL_writing 0.493 0.072 6.857 0.000 0.493 0.495

.max_trial 0.862 0.086 9.975 0.000 0.862 0.866

Community 0.765 0.107 7.176 0.000 1.000 1.000

School 0.759 0.112 6.789 0.000 1.000 1.000

Holiday 0.626 0.097 6.491 0.000 1.000 1.000

Literacy 0.678 0.108 6.297 0.000 1.000 1.000

Home 1.529 0.320 4.777 0.000 1.000 1.000

.Proficiency 0.250 0.062 4.057 0.000 0.485 0.485

R-Square:

Estimate

HL_frnds_t_ch_ 0.769

HL_chld_t_frn_ 0.819

HL_dlts_t_chl_ 0.702

HL_chld_t_dlt_ 0.776

HL_tchr_t_chld 0.763

HL_chld_t_tchr 0.795

HL_frnds_t_chl 0.903

HL_chld_t_frnd 0.931

HL_dlts_t_chl_ 0.629

HL_chld_t_dlt_ 0.778

HL_chldrn_t_c_ 0.723

HL_chld_t_chl_ 0.765

HL_overhrdspch 0.265

SL_onset 0.174

freq_writng_HL 0.682

freq_homwrk_HL 0.665

frq_tschlls_HL 0.030

frq_schllss_HL 0.330

HL_dlt1_t_chld NA

HL_chld_t_dlt1 NA

HL_SES_cargvr1 0.263

HL_speaking 0.518

HL_understndng 0.298

HL_reading 0.475

HL_writing 0.505

max_trial 0.134

Proficiency 0.515
